# Qualitative Analysis of a Mathematical Model in the Time of COVID-19

**DOI:** 10.1155/2020/5098598

**Published:** 2020-05-25

**Authors:** Kamal Shah, Thabet Abdeljawad, Ibrahim Mahariq, Fahd Jarad

**Affiliations:** ^1^Department of Mathematics, University of Malakand, Khyber Pakhtunkhwa, Pakistan; ^2^Department of Mathematics and General Sciences, Prince Sultan University, Riyadh, Saudi Arabia; ^3^Department of Medical Research, China Medical University, Taichung 40402, Taiwan; ^4^Department of Computer Science and Information Engineering, Asia University, Taichung, Taiwan; ^5^College of Engineering and Technology, American University of the Middle East, Kuwait; ^6^Department of Mathematics, Çankaya University, Ankara 06790, Turkey

## Abstract

In this article, a qualitative analysis of the mathematical model of novel corona virus named COVID-19 under nonsingular derivative of fractional order is considered. The concerned model is composed of two compartments, namely, healthy and infected. Under the new nonsingular derivative, we, first of all, establish some sufficient conditions for existence and uniqueness of solution to the model under consideration. Because of the dynamics of the phenomenon when described by a mathematical model, its existence must be guaranteed. Therefore, via using the classical fixed point theory, we establish the required results. Also, we present the results of stability of Ulam's type by using the tools of nonlinear analysis. For the semianalytical results, we extend the usual Laplace transform coupled with Adomian decomposition method to obtain the approximate solutions for the corresponding compartments of the considered model. Finally, in order to support our study, graphical interpretations are provided to illustrate the results by using some numerical values for the corresponding parameters of the model.

## 1. Introduction

Mathematical models are powerful tools to study various physical phenomena of real world problems. The respective idea was initiated by Bernouli in 1776. After that, the first mathematical model of infectious disease was formulated in 1927 by Mckendrick and Karmark. Following that, this area got considerable attention and lots of models, which describe numerous physical or biological processes, were formed; the reader may refer to [1–5] for more information about some models. By using mathematical models for the description of infectious diseases, we can get information about the transmission of a disease in a community, its mortality rates, and how to control it. Therefore, this area has been established in the last few decades very well, (see [6–9]). Several outbreaks in the form of pandemics have been come out like in 1920 and 1967 in which more than 100 million people died. During the start of this century, there also occurred some outbreaks in Saudi Arabia, China, and Mexico. In these outbreaks, thousands of people lost their lives. But due to the rapid advancement in medical science, vaccines were prepared and made those diseases curable. In the end of 2019, a serious outbreak has occurred in Hubei Province of China due to a virus known as corona which has been named the novel COVID-19. This outbreak is in progress and more than three million people have been infected in almost every country of the globe. Nearly 0.22 million people have died due this disease. Three months have gone but, up to date, neither proper cure nor some suitable vaccine have been prepared yet [10]. The World Health Organization (WHO) reported the presence of a novel coronavirus (2019-nCoV) in Wuhan City, Hubei Province of China, on 31 December 2019. The virus which caused this infection belongs to the previous family of SARS. Investigating the present literature, there are many theories behind the origin of the virus. Some researchers have investigated that it originated from bats to human due unlawful full transmissions of animals in a market of seafood in Wuhan. The concerned virus has been identified in pangolin and also in dogs. Therefore, it has been considered that many infected cases claimed that they had been working in a local fish and wild animal market in Wuhan from where they caught infection of coronavirus-19. After that, researchers confirmed that the widespread of the disease is due to person-to-person contact. Also, the mentioned city of the Republic of China is a great trade center from where the infection was transferred to many countries of the world through immigration; for details, we refer to [11, 12].

Keeping in mind what we have mentioned above, numerous researchers started to model the disease to figure out the properties in different ways. Recently, some researchers in [13] have constructed the following mathematical models under ordinary derivative, as a modification to some previously studied prey-predator models [14–16], as follows:

(1) $$\begin{array}{c} \left\{\begin{array}{c} \frac{dH}{dt}=\alpha H\left(t\right)-\beta H\left(t\right)I\left(t\right)+\rho I\left(t\right),\\ \frac{dI}{dt}=\beta H\left(t\right)I\left(t\right)+\gamma I\left(t\right)-\delta I\left(t\right)-\rho I\left(t\right),\\ H\left(0\right)=H_{0},\;I\left(0\right)=I_{0}. \end{array}\right. \end{array}$$

In the above model, *H* stands for healthy individual, *I* for infected individuals, and *β* for the infection rate, where the rate of immigration of healthy individuals from one place to another place is denoted by *α*. Further, the rate at which infection take immigration is *γ*, while death rate is denoted by *δ* and cure rate by *ρ*. Since immigration of people is also a big cause of spreading of this disease, it is evident to check the impact of the immigration of individuals on the transmission dynamics of the current disease. On the other hand, such a study may help in forming some precautionary measures to protect more people from this infection.

The study of the mathematical models under fractional derivatives instead of usual ordinary derivatives produces more significant results which are more helpful in understanding. In fact, numerous fractional order derivatives have been introduced and used in literature including “Caputo and Riemann-Liouville” derivatives which are the most popular differential operators. There are large number of applications in real world problems due to fractional calculus; see [17–23]. Recently, some authors replaced the singular kernel in classical nonlocal fractional derivatives by a nonsingular kernel of “Mittag-Leffler” type; for details, see [24, 25] and the references therein. It is remarkable that fractional derivatives in fact are defined by means of convolutions which contain ordinary derivatives as a special case. Further, the geometry of fractional derivatives tells us about the accumulation of the whole function. Actually, fractional operators, either of singular or without singular kernels, are nonlocal with memory effect unlike ordinary differential operators which are local in nature. Fractional order operators involving “Mittag-Leffler” kernels have been proved more practical and efficient like the classical nonlocal fractional operators; see [25–29]. Further investigating dynamics problems under fractional derivatives instead of integer order derivative produces global dynamics of the concerned problems which include the integer order derivative as a special case [30–36].

Inspired from the aforesaid discussion, in this paper, we investigate the COVID-19 model (1) under the new type derivatives as

(2) DABC0θHt=αHt−βHtIt+pIt,DABC0θIt=βHtIt+γIt−δIt−pIt,H0=H0, I0=I0,

where, in the above model, ^ABC^*𝔻*_0_^*θ*^ stands for Atangana-Baleanu-Caputo (ABC) derivative of order 1 > *θ* > 0. We shall analyze the above model from various aspects including existence theory and series type solution. We shall also investigate some stability results of Ulam's type for the considered model. For the existence theory, we use the classical fixed point theorems of Krasnoselskii's and Banach. In addition to the series type solutions, we shall use the integral transforms given by Laplace and decomposition technique of Adomian. Numerical interpretations are given via graphs to demonstrate the obtained results. Also, it is necessary that the right hand side of the above COVID-19 ABC-model must vanish at 0 (see Theorem 3.1 in [30]).

### 1.1. Organization of the Paper

Section 1 is devoted to introduction of the paper. In Section 2, some fundamental results are given. Also, in Section 3, we establish the existence results while in Section 4, the required analytical results are constructed.  Section 5 is related to the graphical presentations of the results and their discussion. In Section 6, we provide a brief conclusion and some future directions.

## 2. Background Results

Here, we provide some necessary results that may be found in [29] and the references therein such as [24, 25].

**Definition 1 .**

*Ifφ* ∈ *H*1(0, *T*)*andθ* ∈ (0, 1]*, then the ABC derivative is defined by*

(3) DABC0θϕt=κθ1−θ∫0tddyϕyEθ−θ1−θt−yθdy.

*We remark that if we replaceE*_*θ*_[(−*θ*/1 − *θ*)(*t* − *y*)^*θ*^]*byE*_1_ = exp[(−*θ*/1 − *θ*)(*t* − *y*)], *then we get the so-called Caputo-Fabrizo differential operator. Further, it is to be noted that*

(4) DABC0θConstant=0.

*Here,K*(*θ*)*is known as the normalization function which is defined asK*(0) = *K*(1) = 1*. Also,E*_*θ*_*stands for famous special function called Mittag-Leffler which is a generalization to the exponential function* [17–19].

**Definition 2 .**

*Let* *φ* ∈ *L*[0, *T*]*, then the corresponding integral in ABC sense is given by*

(5) IAB0θϕt=1−θKθϕt+θKθΓθ∫0tt−yθ−1ϕydy.

**Lemma 1 . (See Proposition 3 in [28]).**

*The solution of the given problem for*1 > *θ* > 0,

(6) DABC0θϕt=ψt, t∈0,T, ϕ0=ϕ0

is provided by

(7) $$\begin{array}{c} \phi \left(t\right)=\phi_{0}+\frac{\left(1-\theta \right)}{K\left(\theta \right)}\psi \left(t\right)+\frac{\theta }{\Gamma \left(\theta \right)K\left(\theta \right)}\int_{0}^{t}{\left(t-y\right)}^{\theta -1}\psi \left(y\right)dy. \end{array}$$

**Definition 3 .**

*The Laplace transform of ABC derivative of a functionφ*(*t*)*is defined by*

(8) ℒDABC0θϕt=Kθsθ1−θ+θsθℒϕt−sθ−1ϕ0.

*Note:* for the qualitative analysis, we define Banach space as *Z* = *X* × *X*, where *X* = *C*[0, *T*] under the norm  ‖(*H*, *I*)‖ = max_*t*∈[0, *T*]_[|*H*(*t*)+∣*I*(*t*)|]. The following fixed point theorem will be used to proceed to our main results.

**Theorem 1 . (36).**

*LetBbe a convex subset ofZand assume thatF* and *Gare two operators with*

1. *F*(*H*, *I*) + *G*(*H*, *I*) ∈ *Bfor everyH*, *I* ∈ *B*
2. F is contraction
3. G is continuous and compact

*Then, the operator equationF*(*H*, *I*) + *G*(*H*, *I*) = (*H*, *I*)*has at least one solution.*

## 3. Qualitative Analysis of Model (2)

Here, we are going to discuss existence and uniqueness of solution for our main model. Let us write model (2) as

(9) DABC0θHt=ft,Ht,It,DABC0θIt=gt,Ht,It,H0=H0,I0=I0,

where if we apply the fractional integral ^AB^*𝕀*_0_^*θ*^ of order *θ* on both sides of (9) and make use of Lemma 2.3 together with the use of the initial conditions, we get

(10) $$\begin{array}{c} \left\{\begin{array}{c} H\left(t\right)=H_{0}+\frac{\left(1-\theta \right)}{K\left(\theta \right)}f\left(t,H\left(t\right),I\left(t\right)\right)+\frac{\theta }{K\left(\theta \right)\Gamma \left(\theta \right)}\int_{0}^{t}{\left(t-y\right)}^{\theta -1}f\left(y,H\left(y\right)I\left(y\right)\right)dy,\\ I\left(t\right)=I_{0}+\frac{\left(1-\theta \right)}{K\left(\theta \right)}g\left(t,H\left(t\right),I\left(t\right)\right)+\frac{\theta }{K\left(\theta \right)\Gamma \left(\theta \right)}\int_{0}^{t}{\left(t-y\right)}^{\theta -1}g\left(y,H\left(y\right)I\left(y\right)\right)dy. \end{array}\right. \end{array}$$

To derive the existence and uniqueness, we imposed some growth conditions on the nonlinear functions *f*, *g* : [0, *T*] × *R* × *R*⟶*R*.

(A1). There exists constants **L**_*f*_, **L**_*g*_ > 0 such that for each $H,H,\hat{H},\bar{I}\in R$ such that

(11) $$\begin{array}{c} \left|f\left(t,H\left(t\right),I\left(t\right)\right)-f\left(t,\bar{H}\left(t\right),\bar{I}\left(t\right)\right)\right|-\leq L_{f}\left[\left|H-\bar{H}\right|+\left|I-\bar{I}\right|\right],\\ \left|g\left(t,H\left(t\right),I\left(t\right)\right)-g\left(t,\bar{H}\left(t\right),\bar{I}\left(t\right)\right)\right|\leq L_{g}\left[\left|H-\bar{H}\right|+\left|I-\bar{I}\right|\right]. \end{array}$$

(A2). There exists constants *C*_*f*_, *C*_*g*_, *D*_*f*_, *D*_*g*_ > 0 and **M**_*f*_, **M**_*g*_ > 0 such that

(12) $$\begin{array}{c} \left|f\left(t,H\left(t\right),I\left(t\right)\right)\right|\leq C_{f}\left|H\right|+D_{f}\left|H\right|+M_{f}, \end{array}$$

(13) $$\begin{array}{c} \left|g\left(t,H\left(t\right),I\left(t\right)\right)\right|\leq C_{g}\left|H\right|+D_{g}\left|I\right|+M_{g}. \end{array}$$

**Theorem 2 .**

*Under the continuity off*, *gtogether with assumption(A2), system* (5) *has at least one solution if*(1 − 0/*K*(*θ*))**L** < 1*, where ***L** = max{**L**_*f*_, **L**_*g*_}.

**Proof**

By the help of Krasnoselskii's fixed point theorem, we shall prove the existence result. We define the operators *F* = (*F*_1_, *F*_2_), *G* = (*G*_1_, *G*_2_) by using (6) as follows:

(14) $$\begin{array}{c} \left\{\begin{array}{c} F_{1}\left(H,I\right)\left(t\right)=h_{0}+\frac{1-\theta }{K\left(\theta \right)}f\left(t,H\left(t\right),I\left(t\right)\right),\\ G_{1}\left(H,I\right)\left(t\right)=\frac{\theta }{K\left(\theta \right)}\int_{0}^{t}{\left(t-y\right)}^{\theta -1}f\left(y,H\left(y\right),I\left(y\right)\right)dy,\\ F_{2}\left(H,I\right)\left(t\right)=I_{0}+\frac{1-\theta }{K\left(\theta \right)}g\left(t,H\left(t\right),I\left(t\right)\right),\\ G_{2}\left(H,I\right)\left(t\right)=\frac{\theta }{\Gamma \left(\theta \right)}\int_{0}^{t}{\left(t-y\right)}^{\theta -1}g\left(y,H\left(y\right),I\left(y\right)\right)dy. \end{array}\right. \end{array}$$

Now, we show that *F* is a contraction and *G* is a completely continuous operator. For any $\left(H,I\right),\left(\bar{H},\bar{I}\right)\in B,$ we have

(15) $$\begin{array}{c} \left|F_{1}\left(H,I\right)\left(t\right)-F_{1}\left(\bar{H},\bar{I}\right)\left(t\right)\right|\leq \frac{1-\theta }{K\left(\theta \right)}L_{f}\left[\left|H-\bar{H}\right|+\left|I-\bar{I}\right|\right], \end{array}$$

which implies that

(16) $$\begin{array}{c} \left\|F_{1}\left(H,I\right)-F_{1}\left(\bar{H},\bar{I}\right)\right\|\leq \frac{1-\theta }{K\left(\theta \right)}L_{f}\left[\left\|H-\bar{H}\right\|+\left\|I-\bar{I}\right\|\right], \end{array}$$

and similarly, one has

(17) $$\begin{array}{c} \left\|F_{2}\left(H,I\right)-F_{2}\left(\hbar H,\bar{I}\right)\right\|\leq \frac{1-\theta }{K\left(\theta \right)}L_{g}\left[\left\|H-\bar{H}\right\|+\left\|I-\bar{I}\right\|\right]. \end{array}$$

From (8) and (9), one has

(18) $$\begin{array}{c} \left\|F\left(H,I\right)-F\left(\bar{H},\bar{I}\right)\right\|\leq \frac{1-\theta }{K\left(\theta \right)}\left\|\left(H,I\right)-\left(\bar{H},\bar{I}\right)\right\|. \end{array}$$

which implies that *F* is a contraction. Let us define a closed subset *B* of *Z* as

(19) $$\begin{array}{c} B=\left\{\left(H,I\right)\in Z:\;|\;\left(H,I\right)\left\|\leq r,r>0\right.\right\}. \end{array}$$

For *G* to be compact and continuous, let any (*H*, *I*) ∈ *B*, we have

(20) $$\begin{array}{c} \left\|G_{1}\left(H,I\right)\right\|\underset{t\in \left[0,T\right]}{\max}\left|\frac{\theta }{K\left(\theta \right)\Gamma \left(\theta \right)}\int_{0}^{t}{\left(t-y\right)}^{\theta -1}f\left(y,H\left(y,\right)I\left(y,\right)\right)dy\right|\leq \frac{T^{\theta }}{K\left(\theta \right)\Gamma \left(\theta \right)}\left[C_{f}\left\|H\right\|+D_{f}\left\|I\right\|+M_{f}\right],\\ \left\|G_{2}\left(H,I\right)\right\|\underset{t\in \left[0,T\right]}{\max}\left|\frac{\theta }{K\left(\theta \right)\Gamma \left(\theta \right)}\int_{0}^{t}{\left(t-y\right)}^{\theta -1}g\left(y,H\left(y\right),I\left(y\right)\right)dy\right|\leq \frac{T^{\theta }}{K\left(\theta \right)}\left[C_{g}\left\|H\right\|+D_{g}\left\|I\right\|+M_{g}\right]. \end{array}$$

From (10) and (10), we have

(21) $$\begin{array}{c} \left\|G\left(H,I\right)\right\|\leq \frac{T^{\theta }\left(\left[C_{f}+C_{g}+D_{f}+D_{g}\right]r+M_{f}+M_{g}\right)}{K\left(\theta \right)\Gamma \left(\theta \right)}≕\Delta . \end{array}$$

Hence, *F* is bounded. Next, we show that *F* is equicontinuous. Let  *t*_1_ < *t*_2_ ∈ [0, *T*], then consider

(22) $$\begin{array}{c} \left|G_{1}\left(H,I\right)\left(t_{2}\right)-G_{1}\left(H,I\right)\left(t_{1}\right)\right|=\frac{\theta }{K\left(\theta \right)\Gamma \left(\theta \right)}\times \left|\int_{0}^{t_{2}}{\left(t_{2}-y\right)}^{\theta -1}f\left(y,H\left(y\right),I\left(y\right)\right)dy-\int_{0}^{t_{1}}{\left(t_{1}-y\right)}^{\theta -1}f\left(y,H\left(y\right),I\left(y\right)\right)dy\right|\leq \frac{\theta }{K\left(\theta \right)\Gamma \left(\theta \right)}\left[\int_{0}^{t_{2}}{\left(t_{2}-y\right)}^{\theta -1}-\int_{0}^{t_{1}}{\left(t_{1}-y\right)}^{\theta -1}\right]\left(\left(C_{f}+D_{f}\right)r+M_{f}\right)ds\leq \frac{\left(\left(C_{f}+D_{f}\right)r+M_{f}\right)}{K\left(\theta \right)\Gamma \left(\theta \right)}\left[t_{2}^{\theta }-t_{1}^{\theta }\right]. \end{array}$$

Similarly,

(23) $$\begin{array}{c} \left|G_{2}\left(H,I\right)\left(t_{2}\right)-G_{2}\left(H,I\right)\left(t_{1}\right)\right|\leq \frac{\left(\left(C_{g}+D_{g}\right)r+M_{g}\right)}{K\left(\theta \right)\Gamma \left(\theta \right)}\left[t_{2}^{\theta }-t_{1}^{\theta }\right]. \end{array}$$

Now, from (12) and (14), we see as *t*_1_⟶*t*_2_, then the right sides tend to zero. Hence, we see that

(24) $$\begin{array}{c} \left\|G_{1}\left(H,I\right)\left(t_{2}\right)-G_{1}\left(H,I\right)\left(t_{1}\right)\right\|⟶0\text{ and }\left\|G_{1}\left(H,I\right)\left(t_{2}\right)-G_{1}\left(H,I\right)\left(t_{1}\right)\right\|⟶0,\text{ as }t_{1}⟶t_{2}. \end{array}$$

Consequently, we claim that

(25) $$\begin{array}{c} \left\|G\left(H,I\right)\left(t_{2}\right)-G\left(H,I\right)\left(t_{1}\right)\right\|⟶0,\text{ as }t_{1}⟶t_{2}. \end{array}$$

Hence, *G* is a equicontinuous operator. By using Arzelà–Ascoli theorem, the operator *G* is a completely continuous operator and also uniformly bounded proved already. Hence, *G* is relatively compact. By Krasnoselskii's fixed point theorem, the given system has at least one solution.

Next, we establish results about uniqueness of solution as follows:

**Theorem 3 .**

*Under the assumption(A1), our COVID-19 system under ABC derivative has a unique solution if*

(26) $$\begin{array}{c} \frac{2T^{\theta }}{K\left(\theta \right)\Gamma \left(\theta \right)}L<1, \end{array}$$

*with*max{**L**_*f*_, **L**_*g*_} = **L**.

**Proof**

Define the operator **P** = (**P**_1_, **P**_2_): *Z*⟶*Z* using (6) as

(27) $$\begin{array}{c} \left\{\begin{array}{c} P_{1}\left(H,I\right)\left(t\right)=H_{0}+\frac{\left(1-\theta \right)}{K\left(\theta \right)}f\left(t,H\left(t\right),I\left(t\right)\right)+\frac{\theta }{K\left(\theta \right)\Gamma \left(\theta \right)}\int_{0}^{t}{\left(t-y\right)}^{\theta -1}f\left(y,H\left(y,\right)I\left(y,\right)\right)dy,\\ P_{2}\left(H,I\right)\left(t\right)=I_{0}+\frac{\left(1-\theta \right)}{K\left(\theta \right)}g\left(t,H\left(t\right),I\left(t\right)\right)+\frac{\theta }{K\left(\theta \right)\Gamma \left(\theta \right)}\int_{0}^{t}{\left(t-y\right)}^{\theta -1}g\left(y,H\left(y,\right)I\left(y,\right)\right)dy. \end{array}\right. \end{array}$$

Now, we take (*H*, *I*) and $\left(\bar{H},\bar{I}\right)$ ∈ *Z* and using (15), we have

(28) $$\begin{array}{c} \left\|P_{1}\left(H,I\right)-P_{1}\left(\bar{H},\bar{I}\right)\right\|=\underset{t\in \left[0,T\right]}{\max}\left|\frac{\theta }{K\left(\theta \right)\Gamma \left(\theta \right)}\int_{0}^{t}{\left(t-y\right)}^{\theta -1}\left[f\left(y,H\left(y,\right)I\left(y,\right)\right)-f\left(y,\bar{H}\left(y\right),\bar{I}\left(y,\right)\right)dy\right.\right|\leq \frac{T^{\theta }}{K\left(\theta \right)\Gamma \left(\theta \right)}L_{f}\left[\left\|H-\bar{H}\right\|+\left\|I-\bar{I}\right\|\right], \end{array}$$

and in same fashion, one has

(29) $$\begin{array}{c} \left\|P_{2}\left(H,I\right)-P_{2}\left(\bar{H},\bar{I}\right)\right\|\leq \frac{T^{\theta }}{K\left(\theta \right)\Gamma \left(\theta \right)}L_{f}\left[\left\|H-\bar{H}\right\|+\left\|I-\bar{I}\right\|\right]. \end{array}$$

From (16) and (17), we have

(30) $$\begin{array}{c} \left\|P\left(H,I\right)-P\left(\bar{H},\bar{I}\right)\right\|\leq \frac{2T^{\theta }}{K\left(\theta \right)\Gamma \left(\theta \right)}L\left\|\left(H,I\right)-\left(\bar{H},\bar{I}\right)\right\|. \end{array}$$

Hence, **P** is a contraction. By Banach contraction theorem, the considered system has unique solution.

Next, we give a results about Ulam-Hyers stability.

**Theorem 4 .**

*The solution of the considered model* (2) *is Ulam-Hyers stable if the spectral radius of the following matrix*

(31) $$\begin{array}{c} \left[\begin{array}{cc} a & a\\ b & b \end{array}\right], \end{array}$$

*given by*|**a** + **b**| < 1*, where*

(32) $$\begin{array}{c} a=\left(\frac{\left(1-\theta \right)}{K\left(\theta \right)}+\frac{T^{\theta }}{K\left(\theta \right)\Gamma \left(\theta \right)}\right)L_{f,}\\ b=\left(\frac{\left(1-\theta \right)}{K\left(\theta \right)}+\frac{T^{\theta }}{K\left(\theta \right)\Gamma \left(\theta \right)}\right)L_{g}. \end{array}$$

**Proof**

*Let* (*H*, *I*) ∈ *Z* be any solution of the model (2) and $\left(\bar{H},\bar{I}\right)\in Z$ is unique solution of the same model; then, we have

(33) $$\begin{array}{c} \left\{\left\|\left(H,I\right)-\left(\bar{H},\bar{I}\right)\right\|\right.\leq \left[\begin{array}{cc} a & a\\ b & b \end{array}\right]\left[\begin{array}{c} \left\|H-\bar{H}\right\|\\ \left\|I-\bar{I}\right\| \end{array}\right], \end{array}$$

where **a** and **b** are given as in (19). Hence, the solution of the given system is Ulam-Hyers stable. Since the eigenvalues of square matrix are  *λ*_1_ = 0, *λ*_2_ = **a** + **b** and spectral radius of the matrix is given by max{|*λ*1|: *i* = 1, 2} = |**a** + **b**| < 1.

## 4. Analytical Study of Model (2)

In this section, we apply the proposed novel analytical method to find the series type solution of the suggested model (2). To this end, we take the Laplace transform of both sides of (2) and use the initial conditions to get

(34) $$\begin{array}{c} \left\{\begin{array}{c} \frac{K\left(\theta \right)}{\left[s^{\theta }\left(1-\theta \right)+\theta \right]}\left[s^{\theta }ℒ\left[H\left(t\right)\right]-s^{\theta -1}H_{0}\right]=ℒ\left[\alpha H\left(t\right)-\beta H\left(t\right)I\left(t\right)+\rho I\left(t\right)\right],\\ \frac{K\left(\theta \right)}{\left[s^{\theta }\left(1-\theta \right)+\theta \right]}\left[s^{\theta }ℒ\left[I\left(t\right)\right]-s^{\theta -1}I_{0}\right]=ℒ\left[\beta H\left(t\right)I\left(t\right)+\gamma I\left(t\right)-\delta I\left(t\right)-\rho I\left(t\right)\right]. \end{array}\right. \end{array}$$

After rearranging the terms in (34), one has

(35) $$\begin{array}{c} \left\{\begin{array}{c} ℒ\left[H\left(t\right)\right]=\frac{H_{0}}{s}+\frac{\left[s^{\theta }\left(1-\theta \right)+\theta \right]}{s^{\theta }K\left(\theta \right)}ℒ\left[\alpha H\left(t\right)-\beta H\left(t\right)I\left(t\right)+\rho I\left(t\right)\right],\\ ℒ\left[I\left(t\right)\right]=\frac{I_{0}}{s}+\frac{\left[s^{\theta }\left(1-\theta \right)+\theta \right]}{s^{\theta }K\left(\theta \right)}ℒ\left[\beta H\left(t\right)I\left(t\right)+\gamma I\left(t\right)-\delta I\left(t\right)-\rho I\left(t\right)\right]. \end{array}\right. \end{array}$$

Now, we are interested to find the required solution in the form of infinite series, therefore taking the unknown solutions

(36) $$\begin{array}{c} H\left(t\right)=\overset{\infty }{\underset{n=0}{\sum }}H_{n}\left(t\right),\\ I\left(t\right)=\overset{\infty }{\underset{n=0}{\sum }}I_{n}\left(t\right). \end{array}$$

Further, the nonlinear term *H*(*t*)*I*(*t*) in the system (2) may be decomposed in terms of Adomian polynomials as

(37) $$\begin{array}{c} H\left(t\right)I\left(t\right)=\overset{\infty }{\underset{n=0}{\sum }}Q_{n}\left(H,I\right), \end{array}$$

where

(38) $$\begin{array}{c} Q_{n}\left(H,I\right)=\frac{1}{n!}\frac{d^{n}}{d\lambda^{n}}\left[\overset{n}{\underset{i=0}{\sum }}\lambda^{i}H_{i}\left(t\right)\overset{n}{\underset{i=0}{\sum }}\lambda^{i}I_{i}\left(t\right)\right]\left|{}_{\lambda =0}\right.. \end{array}$$

We compute few terms for *n* = 0, 1, 2, ⋯, as

(39) $$\begin{array}{c} Q_{0}\left(H,I\right)=H_{0}\left(t\right)I_{0}\left(t\right),\\ Q_{1}\left(H,I\right)=H_{1}\left(t\right)I_{0}\left(t\right)+H_{0}\left(t\right)I_{1}\left(t\right),\\ Q_{2}\left(H,I\right)=H_{1}\left(t\right)I_{1}\left(t\right)+H_{0}\left(t\right)I_{2}\left(t\right)+H_{2}\left(t\right)I_{0}\left(t\right), \end{array}$$

and so on. Plugging the above series type representation in (21), one has

(40) $$\begin{array}{c} \left\{\begin{array}{c} ℒ\left[\overset{\infty }{\underset{n=0}{\sum }}H_{n}\left(t\right)\right]=\frac{H_{0}}{s}+\frac{\left[s^{\theta }\left(1-\theta \right)+\theta \right]}{s^{\theta }K\left(\theta \right)}ℒ\left[\alpha \overset{\infty }{\underset{n=0}{\sum }}H_{n}\left(t\right)-\beta \overset{\infty }{\underset{n=0}{\sum }}Q_{n}\left(H,I\right)+\rho \overset{\infty }{\underset{n=0}{\sum }}I_{n}\left.\left(t\right)\right)\right],\\ ℒ\left[\overset{\infty }{\underset{n=0}{\sum }}I_{n}\left(t\right)\right]=\frac{I_{0}}{s}+\frac{\left[s^{\theta }\left(1-\theta \right)+\theta \right]}{s^{\theta }K\left(\theta \right)}ℒ\left[\beta \overset{\infty }{\underset{n=0}{\sum }}Q_{n}\left(H,I\right)+\gamma \overset{\infty }{\underset{n=0}{\sum }}I_{n}\left(t\right)\left(t\right)-\delta \overset{\infty }{\underset{n=0}{\sum }}I_{n}\left(t\right)\left(t\right)-\rho \overset{\infty }{\underset{n=0}{\sum }}I_{n}\left(t\right)\left(t\right)\right]. \end{array}\right. \end{array}$$

Comparing terms on both sides in (40), we get

(41) $$\begin{array}{c} \left\{\begin{array}{c} ℒ\left[H_{0}\left(t\right)\right]=\frac{H_{0}}{s},\;ℒ\left[I_{0}\left(t\right)\right]=\frac{I_{0}}{s},\\ ℒ\left[H_{1}\left(t\right)\right]=\frac{\left[s^{\theta }\left(1-\theta \right)+\theta \right]}{s^{\theta }K\left(\theta \right)}ℒ\left[\alpha H_{0}\left(t\right)-\beta Q_{0}\left(H,I\right)+\rho I_{0}\left.\left(t\right)\right)\right],\\ ℒ\left[I_{1}\left(t\right)\right]=\frac{\left[s^{\theta }\left(1-\theta \right)+\theta \right]}{s^{\theta }K\left(\theta \right)}ℒ\left[\beta Q_{0}\left(H,I\right)+\gamma I_{0}\left(t\right)-\delta I_{0}\left(t\right)\left(t\right)-\rho I_{0}\left(t\right)\left(t\right)\right],\\ \vdots \\ ℒ\left[H_{n+1}\left(t\right)\right]=\frac{\left[s^{\theta }\left(1-\theta \right)+\theta \right]}{s^{\theta }K\left(\theta \right)}ℒ\left[\alpha H_{n}\left(t\right)-\beta Q_{n}\left(H,I\right)+\rho I_{n}\left.\left(t\right)\right)\right],\\ ℒ\left[I_{n+1}\left(t\right)\right]=\frac{\left[s^{\theta }\left(1-\theta \right)+\theta \right]}{s^{\theta }K\left(\theta \right)}ℒ\left[\beta Q_{n}\left(H,I\right)+\gamma I_{n}\left(t\right)-\delta I_{n}\left(t\right)\left(t\right)-\rho I_{n}\left(t\right)\left(t\right)\right],\;n\geq 0. \end{array}\right. \end{array}$$

Taking inverse transform of Laplace on both sides and after computation with using

(42) $$\begin{array}{c} \Delta_{1}=\frac{\left(\alpha H_{0}-\beta H_{0}I_{0}+\rho I_{0}\right)}{K\left(\theta \right)},\\ \Delta_{2}=\frac{\left(\rho H_{0}I_{0}+\left(\gamma -\delta -\rho \right)I_{0}\right)}{K\left(\theta \right)}, \end{array}$$

we get few terms of the series solution as

(43) $$\begin{array}{c} \left\{\begin{array}{c} H_{0}\left(t\right)=H_{0},\;I_{0}\left(t\right)=I_{0},\\ H_{1}\left(t\right)=\Delta_{1}\left[1-\theta +\frac{t^{\theta }}{\Gamma^{\theta }}\right],\\ I_{1}\left(t\right)=\Delta_{2}\left[1-\theta +\frac{t^{\theta }}{\Gamma^{\theta }}\right],\\ H_{2}\left(t\right)=\frac{\left[\alpha \Delta_{1}-\beta \left(H_{0}\Delta_{2}+I_{0}\Delta_{1}\right)+\rho \Delta_{2}\right]}{K\left(\theta \right)}\left[{\left(1-\theta \right)}^{2}+\frac{2\left(1-\theta \right)t^{\theta }}{\Gamma \left(\theta \right)}+\frac{\theta t^{2\theta }}{2\Gamma \left(2\theta \right)}\right],\\ I_{2}\left(t\right)=\frac{\left[\beta \left(\Delta_{1}I_{0}+H_{0}\Delta_{2}\right)+\left(\gamma -\delta -\rho \right)\Delta_{2}\right]}{K\left(\theta \right)}\left[{\left(1-\theta \right)}^{2}+\frac{2\left(1-\theta \right)t^{\theta }}{\Gamma \left(\theta \right)}+\frac{\theta t^{2\theta }}{2\Gamma \left(2\theta \right)}\right],\\ H_{3}\left(t\right)=\frac{H_{0}\left[\beta \left(\Delta_{1}I_{0}+H_{0}\Delta_{2}\right)+\left(\gamma -\delta -\rho \right)\Delta \right]+I_{0}\left[\alpha \Delta -\beta \left(H_{0}\Delta_{2}+I_{0}\Delta_{1}\right)+\rho \Delta_{2}\right]+\alpha \left[\alpha \Delta_{1}-\beta \left(H_{0}\Delta_{2}+I_{0}\Delta_{1}\right)+\rho \Delta_{2}\right]}{{\left[K\left(\theta \right)\right]}^{2}}\times \left[{\left(1-\theta \right)}^{3}+\frac{\left[2\left(1-\theta \right)+{\left(1-\theta \right)}^{2}\right]t^{\theta }}{\Gamma \left(\theta \right)}+\frac{\left[5\theta \left(1-\theta \right)\right]t^{2\theta }}{2\Gamma \left(2\theta \right)}+\frac{\left[\theta^{2}\right]t^{3\theta }}{\Gamma \left(3\theta \right)}\right]-\frac{\beta \Delta_{1}\Delta_{2}}{{\left[K\left(\theta \right)\right]}^{2}}\left[{\left(1-\theta \right)}^{3}+2{\left(1-\theta \right)}^{2}\frac{t^{\theta }}{\Gamma \left(\theta \right)}+\left(1-\theta \right)\frac{t^{2\theta }}{\Gamma^{2}\left(\theta \right)}+\frac{\theta \left(1-\theta \right)t^{2\theta }}{\Gamma \left(2\theta \right)}+\frac{\theta \Gamma \left(2\theta +1\right)t^{3\theta }}{\Gamma^{2}\left(\theta \right)\Gamma \left(3\theta +1\right)}\right],\\ I_{3}\left(t\right)=\frac{\beta H_{0}\left[\beta \left(\Delta_{1}I_{0}+H_{0}\Delta_{2}\right)+\left(\gamma -\delta -\rho \right)\Delta_{2}\right]+\beta I_{0}\left[\alpha \Delta_{1}-\beta \left(H_{0}\Delta_{2}+I_{0}\Delta_{1}\right)+\rho \Delta_{2}\right]+\left(\gamma -\delta -\rho \right)\left[\beta \left(\Delta_{1}I_{0}+H_{0}\Delta_{2}\right)+\left(\gamma -\delta -\rho \right)\Delta_{2}\right]}{{\left[K\left(\theta \right)\right]}^{2}}\times \left[{\left(1-\theta \right)}^{3}+\frac{\left[2\left(1-\theta \right)+{\left(1-\theta \right)}^{2}\right]t^{\theta }}{\Gamma \left(\theta \right)}+\frac{\left[5\theta \left(1-\theta \right)\right]t^{2\theta }}{2\Gamma \left(2\theta \right)}+\frac{\left[\theta^{2}\right]t^{3\theta }}{\Gamma \left(3\theta \right)}\right]-\frac{\beta \Delta_{1}\Delta_{2}}{{\left[K\left(\theta \right)\right]}^{2}}\left[{\left(1-\theta \right)}^{3}+2{\left(1-\theta \right)}^{2}\frac{t^{\theta }}{\Gamma \left(\theta \right)}+\left(1-\theta \right)\frac{t^{2\theta }}{\Gamma^{2}\left(\theta \right)}+\frac{\theta \left(1-\theta \right)t^{2\theta }}{\Gamma \left(2\theta \right)}+\frac{\theta \Gamma \left(2\theta +1\right)t^{3\theta }}{\Gamma^{2}\left(\theta \right)\Gamma \left(3\theta +1\right)}\right], \end{array}\right. \end{array}$$

and so on. In this way, the remaining terms will be generated.

## 5. Numerical Simulation and Justification of Qualitative Results

Now, we take some various values for parameters taken in [16] as *α* = 0.0, *β* = 0.03, *γ* = 0.05, *δ* = 0.05, and *ρ* = 0.05 and take a random community where the total population is divided in such a way that 70 percent of the population is healthy and 30 percent is infected, that *H*0 = 0.7, *I* = 0.3. Clearly, using these values in model (2), we have **L**_*f*_ = 0.03, **L**_*g*_ = 0.03, *K*(*θ*) = 1. From which we have **L** = 0.03. Hence, the condition of existence of at least one solution holds by using Theorem 3.1. Also, the condition of Theorem 3.3 is valid under suitable value of *T*. In the current situation, the solution is going to become stable. Further, taking *K*(*θ*) = 1, we compute few terms from (43) of series solution up to four terms as follows:

(44) $$\begin{array}{c} \left\{\begin{array}{c} H\left(t\right)=0.7+0.087\left[1-\theta +\frac{t^{\theta }}{\Gamma \left(\theta \right)}\right]+0.02412\left[{\left(1-\theta \right)}^{2}+\frac{2\left(1-\theta \right)t^{\theta }}{\Gamma \left(\theta \right)}+\frac{\theta t^{2\theta }}{2\Gamma \left(2\theta \right)}\right]+0.008436\left[{\left(1-\theta \right)}^{3}+\frac{\left[2\left(1-\theta \right)+{\left(1-\theta \right)}^{2}\right]t^{\theta }}{\Gamma \left(\theta \right)}+\frac{\left[5\theta \left(1-\theta \right)\right]t^{2\theta }}{2\Gamma \left(2\theta \right)}+\frac{\left[\theta^{2}\right]t^{3\theta }}{\Gamma \left(3\theta \right)}\right]-0.0011745\left[{\left(1-\theta \right)}^{3}+2{\left(1-\theta \right)}^{2}\frac{t^{\theta }}{\Gamma \left(\theta \right)}+\left(1-\theta \right)\frac{t^{2\theta }}{\Gamma^{2}\left(\theta \right)}+\frac{\theta \left(1-\theta \right)t^{2\theta }}{\Gamma \left(2\theta \right)}+\frac{\theta \Gamma \left(2\theta +1\right)t^{3\theta }}{\Gamma^{2}\left(\theta \right)\Gamma \left(3\theta +1\right)}\right],\\ I\left(t\right)=0.3-0.045\left[1-\theta +\frac{t^{\theta }}{\Gamma \left(\theta \right)}\right]-0.02412\left[{\left(1-\theta \right)}^{2}+\frac{2\left(1-\theta \right)t^{\theta }}{\Gamma \left(\theta \right)}+\frac{\theta t^{2\theta }}{2\Gamma \left(2\theta \right)}\right]-0.0190044\left[{\left(1-\theta \right)}^{3}+\frac{\left[2\left(1-\theta \right)+{\left(1-\theta \right)}^{2}\right]t^{\theta }}{\Gamma \left(\theta \right)}+\frac{\left[5\theta \left(1-\theta \right)\right]t^{2\theta }}{2\Gamma \left(2\theta \right)}+\frac{\left[\theta^{2}\right]t^{3\theta }}{\Gamma \left(3\theta \right)}\right]+0.0011745\left[{\left(1-\theta \right)}^{3}+2{\left(1-\theta \right)}^{2}\frac{t^{\theta }}{\Gamma \left(\theta \right)}+\left(1-\theta \right)\frac{t^{2\theta }}{\Gamma^{2}\left(\theta \right)}+\frac{\theta \left(1-\theta \right)t^{2\theta }}{\Gamma \left(2\theta \right)}+\frac{\theta \Gamma \left(2\theta +1\right)t^{3\theta }}{\Gamma^{2}\left(\theta \right)\Gamma \left(3\theta +1\right)}\right], \end{array}\right. \end{array}$$

and so on.

**Figure 1:**
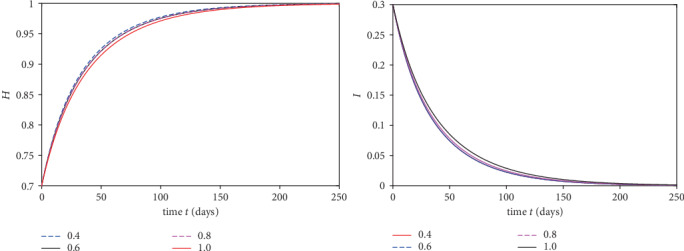
Plot of both classes at different fractional orders and taking immigration rate *α* = 0.0 and *γ* = 0.0.

**Figure 2:**
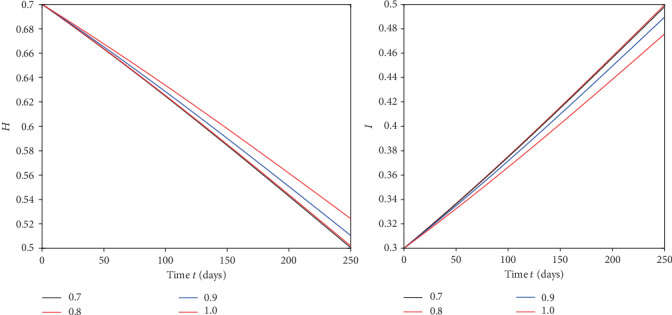
Plot of both classes at different fractional orders under immigration *α* = 0.05 and *γ* = 0.5.

**Figure 3:**
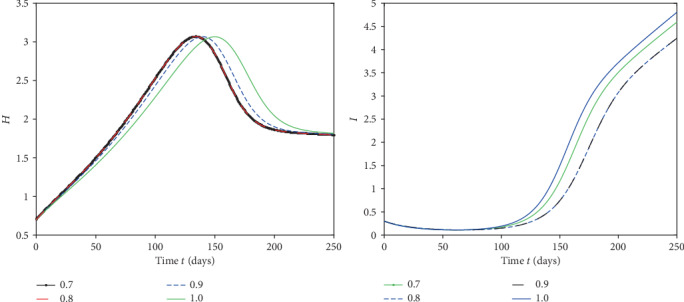
Plot of both classes at different fractional orders and taking immigration rate *α* = 0 and *γ* = 0.05.

**Figure 4:**
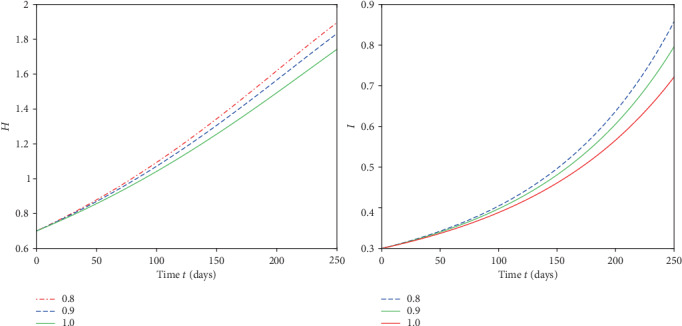
Plot of both classes at different fractional orders and taking immigration rate *α* = 0.05 and *γ* = 0.0.

We plot the solutions (43) for different fractional order by using MATLAB in Figures 1–4.

From Figure 1, we see that at when the rate of healthy immigrants is zero, it means that protection rate is increasing and hence the population of infected class is decreasing while the population of healthy class is increasing at different rates due to fractional order derivative by evaluating the solution up to twenty terms via using MATAB. As the order is increasing, the growth rate of healthy class is increasing and thus becomes stable first as compared to the small fractional order. On the other hand, the decaying process of infected class is fastest on the small fractional order as compared to the large order. Thus, in this case, the stability is achieved first at the smallest fractional order derivative rather than at the greater order. Further at the given values of the parameters, the infection to vanish in a locality will take days between 220 and 250. In Figure 2, in the presence of immigration and less protection rate, we plot the solution corresponding to different fractional orders. We see that infection is increasing while the population of healthy class is decreasing at various rates due to fractional order. From Figure 3, when we involve immigration of infected class and cease the immigration for the healthy class, we see that the population density of infected class is going up with different rates due to fractional order derivative during in first 250 days. On the other hand, the healthy population is going on instability in the first 50 days, That is; it increases and then decreases suddenly. To achieve stable position, it requires nearly 110 days. This means that immigration of infected population from one place to another will cause instability in the healthy population of a community. From Figure 4, we see that the straight increase in both populations is due to immigration of healthy population but using strong protection rate at different fractional orders.

## 6. Conclusion

In this article, we have examined a population model of the novel COVID-19 under *ABC* fractional order derivatives. We have proved sufficient results about the existence and uniqueness of solution for the considered model and proved that it has at least one solution. Hence, the fixed point theory always works as an effective tool that can be used to check the existence and uniqueness of various physical problems. A stability result has also been established. Through a novel method, we have derived approximate solutions for the corresponding compartments of the model under investigation. Further, some numerical results have been presented for different fractional orders through MATLAB by taking various values of the immigration rates. We observed that as the immigration of infected class is increasing, it will cause the decrease in healthy population and hence the population of infected people increases. Therefore, an important factor which increases the infection of the current outbreak is free immigration. When people do not avoid the unnecessary traveling from one place to another, there is greater chance to infect. If this term decreases, then infection may be sufficiently decreased in a population. If in society, the immigration of infected people is strictly controlled, then we may protect our society from further hazard. In the future, the concerned model may be further extended by involving exposed class, recovered class, and asymptotically infected class to form five compartment models. This new model will further produce more significant information basis on which better controlling policies and procedure may be made to save our society from this infection.

## Data Availability

No data were used to support this study.
